# Supplementary material for: Wildlife–Livestock Host Community Maintains Simultaneous Epidemiologic Cycles of Mycoplasma conjunctivae in a Mountain Ecosystem
Source: Vet Sci. 2024 May 14;11(5):217. doi: 10.3390/vetsci11050217 (PMC11125856; doi:10.3390/vetsci11050217)
Supplement: Supplementary file 1 [file vetsci-11-00217-s001.zip › vetsci-2926521-supplementary.pdf]

Table S1. Primers and probes used in this study for *Mycoplasma conjunctivae* detection and Sanger DNA sequence analysis.

**Primers and probes**	**Sequences (5′-3′)**	**Use**
LPPS-TM-L	CAGCTGGTGTAGCACTTTTTGC	qPCR
LPPS-TM-R	TTAACACCTATGCTCTCGTCTTTGA	qPCR
LPPS-TM-FT	TGCTTCGACTACCAAATATGATGGTGATCCTCT[a]	qPCR - probe
Serstart3	TTTAGTAGACTCCACTTCACC	PCR
Serstart2	CACTATACTTAACAGATAGTCC	Nested PCR, Sequencing
Serstart0	ATACTCAAAGTGGAAATAATGGAA	Sequencing
Serend0	GCAACAACAATAGTAAGAGCAG	Sequencing
lppTA2	TTTGATCTCTCCACCTTCAGC	PCR
lppTA	GGCACTAATAGTGCGTAATTC	Nested PCR

[a]5′ 6FAM reporter dye and 3′ TAMRA quencher.
